# Tau interferes with axonal neurite stabilization and cytoskeletal composition independently of its ability to associate with microtubules

**DOI:** 10.1242/bio.052530

**Published:** 2020-09-25

**Authors:** Edward F. Boumil, Rishel B. Vohnoutka, Sangmook Lee, Thomas B. Shea

**Affiliations:** Laboratory for Neuroscience, Department of Biological Sciences, UMass Lowell, Lowell, MA 01854, USA

**Keywords:** Tau, Axonal transport, Axonal neurite, Neurite stabilization, Cytoskeleton, Neurofilament, Microtubules

## Abstract

Tau impacts overall axonal transport particularly when overexpressed by interfering with translocation of kinesin along microtubules (MTs) and/or as a cargo of kinesin by outcompeting other kinesin cargo. To discern between which of these mechanisms was more robust during axonal outgrowth, we overexpressed phosphomimetic (E18; which is incapable of MT binding), phospho-null (A18) or wild-type (WT) full-length human tau conjugated to EGFP, the latter two of which bind MTs. Expression of WT and A18 displayed increased acetylated MTs and resistance to colchicine, while expression of E18 did not, indicating that E18 did not contribute to MT stabilization. Expression of all tau constructs reduced overall levels of neurofilaments (NFs) within axonal neurites, and distribution of NFs along neurite lengths. Since NFs are another prominent cargo of kinesin during axonal neurite outgrowth, this finding is consistent with WT, A18 and E18 inhibiting NF transport to the same extent by competing as cargo of kinesin. These findings indicate that tau can impair axonal transport independently of association with MTs in growing axonal neurites.

## Abstract (precis)

**Summary:** Tau inhibits axonal transport by blocking kinesin translocation or as kinesin cargo. Tau capable and incapable of microtubule binding inhibited transport equally, indicating inhibition of axonal transport as kinesin cargo.

## INTRODUCTION

Microtubules (MTs), cytoskeletal polymers composed of alpha and beta tubulin, provide structural support to axons and mediate the movement of cargo into and out of the axon via the motor proteins kinesin and dynein that traverse along them. Cargoes of these motors include vesicles (Bury and Sabo, 2011), mitochondria (Saxton and Hollenbeck, 2012), and cytoskeletal elements themselves including neurofilaments (NFs; Yabe et al., 2000; Xia et al., 2003; Shah et al., 2000; Motil et al., 2006; Lee et al., 2011; Sunil et al., 2012).

MTs are often decorated with an assortment of microtubule-associated proteins (MAPs), which influence MT stability (Baas et al., 1994; Goold et al., 1999) and link MTs to other components of the cytoskeleton (Hirokawa et al., 1988; Miyasaka et al., 1993). One such MAP, tau, is thought to provide stability to axonal MTs (Lo et al., 1993; de Garcini et al., 1994; Baas et al., 1994), and facilitate MT–MT crosslinking that leads to formation of MT bundles (Kanai et al., 1989; Wagner et al., 1996; Rodríguez-Martín et al., 2013). Tau also induces MT acetylation, which correlates with increased resistance of MTs to depolymerization (Cohen et al., 2013) and increases anterograde axonal transport (Bulinski, 2007). The mechanism underlying the role(s) of tau in MT stabilization remains unclear, since tau is more concentrated along dynamic rather than stabilized MTs within axons (Black et al., 1996) and furthermore within labile rather than stable MT domains (Qiang et al., 2018). Tau may indirectly contribute to MT stabilization by increasing the length of MTs, followed by MT stabilization by other MAPs (Baas and Qiang, 2019).

Tau impacts overall axonal transport by at least two mechanisms (for review, see Talmat-Amar et al., 2018). Tau interferes with translocation of the anterograde motor kinesin along MTs (Dixit et al., 2008; Hoeprich et al., 2017; Stamer et al., 2002; Stern et al., 2017). Tau is also transported along MTs as a cargo of kinesin (Cuchillo-Ibanez et al., 2008; Dubey et al., 2008; Gendron and Petrucelli, 2009; for reviews see Scholz and Mandelkow, 2014 and Pérez et al., 2018) and when in excess can inhibit transport of other cargo by competing for available kinesin (Dubey et al., 2008).

We wished to probe further the relative impact of tau phosphorylation on axonal neurite development. Since this can be confounded by developmental changes in the activity of kinases and phosphatases, we compared wild-type (WT) tau to that of tau pseudophosphorylated at multiple Glycogen-Synthase Kinase-3beta consensus sites and tau unable to be phosphorylated at these sites along with WT tau.

## RESULTS

### Pseudophosphorylation of tau influences association with MTs

**Fig. 1.**
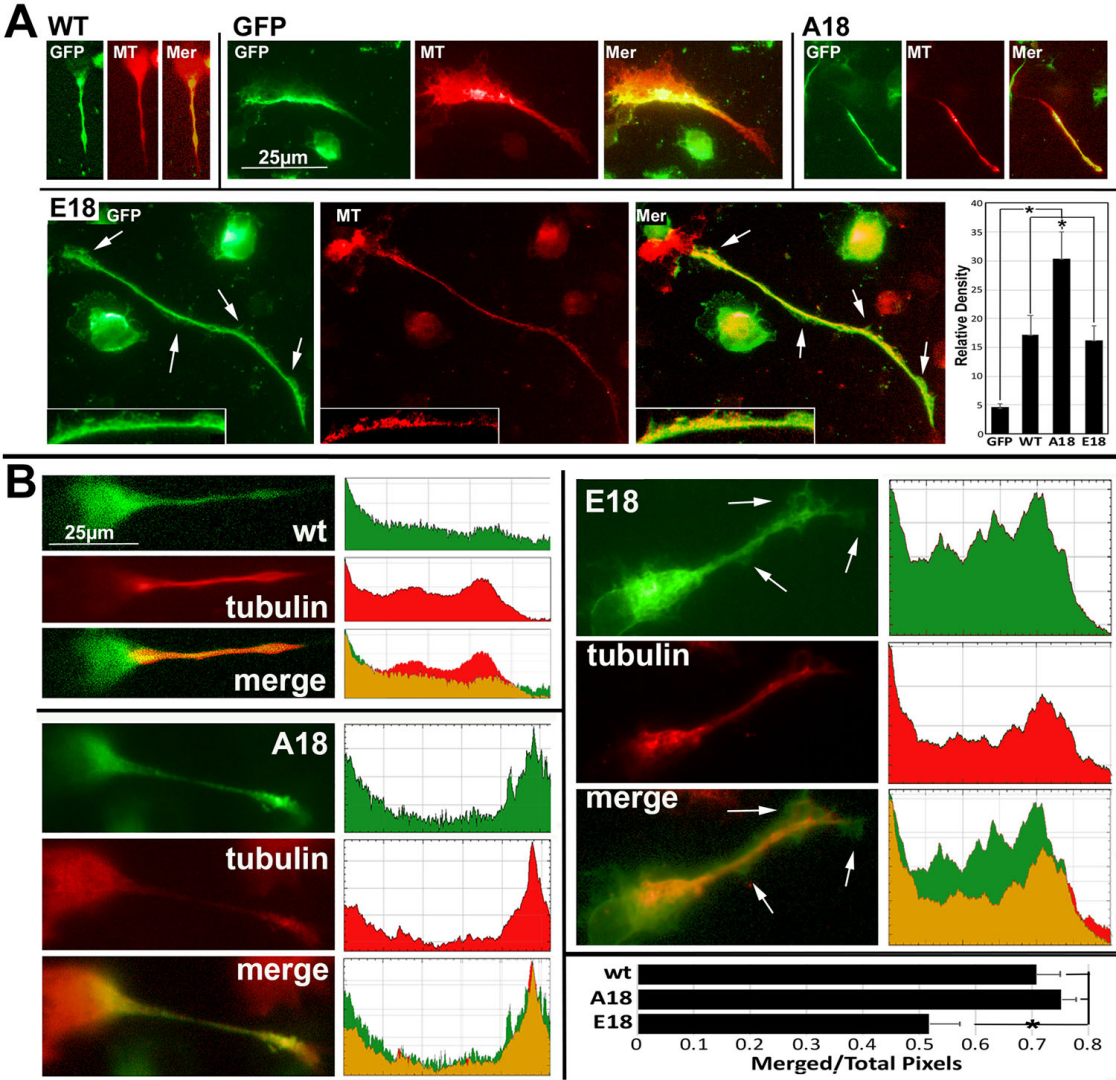
**Pseudophosphorylation of tau influences distribution within neurites.** Panel A presents representative epifluorescence images of GFP, corresponding tubulin immunoreactivity (MT detected by antibody DM1A) and merged images (Mer) for cells transfected 48 h after differentiation with vectors encoding for unconjugated GFP (*n*=76), WT (*n*=65), A18 (*n*=78) and E18 (*N*=64). Note colocalization of most neuritic WT and A18 with MT immunoreactivity but distribution of E18 into the neuritic cytoplasm beyond MT immunoreactivity (arrows indicate some examples; high magnification inserts present the region of the neurite indicated by the two rightmost arrows). Relatively little unconjugated GFP translocated into neurites (*P*<0.001 versus all tau constructs; one-way ANOVA). Identical levels of WT and E18 were distributed within neurites (*P*=0.23). By contrast, increased levels of A1 were distributed within neurites versus WT (*P*=0.04) and E18 (*P*=0.01). All images within panel A and within panel B were of the same magnification. Panel B presents representative epifluorescence images of GFP, corresponding tubulin immunoreactivity (tubulin detected by antibody DM1A) and merged images (merged) along with measurement of fluorescence within neurites (excluding the hillock and growth cone) generated with the ‘plot profile’ feature of ImageJ. As in panel A, WT and A18 colocalized with tubulin immunoreactivity while E18 extended further into the neuritic cytoplasm than tubulin (arrow). The accompanying graph presents the ratio of the area of colocalized GFP and tubulin pixels versus total area containing fluorescent pixels within neurites derived from plot profiles of three cells for each construct. Note identical ratios for WT and A18 but a reduction in colocalization for E18 (asterisk indicates *P*<0.05; one-way ANOVA).

Quantification of GFP levels within soma 24 h after transfection demonstrated statistically equivalent expression of all tau isoforms [WT: 6784.6±2514 (*n*=57), A18: 5658.4±3184.3 (*n*=71), E18 6349.1±2598.6 (*n*=50), mean intensity±standard deviation]; this was anticipated since the identical expression vector was utilized for each isoform. Consistent with prior studies, WT, phospho-null (A18) and phosphomimetic (E18) full-length human tau translocated into and along the entire length of established neurites (Fig. 1). Identical levels of WT and E18 were distributed within neurites. By contrast, increased levels of A18 were distributed within neurites versus WT and E18. Relatively low levels of GFP not conjugated with tau (‘unconjugated GFP’) translocated into neurites, consistent with diffusion of unconjugated GFP versus active transport of GFP-tau conjugates.

Tubulin immunofluorescence was relatively centralized and linear along the length of axonal neurites (Fig. 1A,B). Visual inspection (Fig. 1A) and plot profile analyses (Fig. 1B) indicated that the distribution of WT and A18 largely correlated with that of tubulin within axonal neurites. By contrast, substantial levels of E18 extended into the surrounding cytoplasm and cytoplasmic extensions the along the neurite length, which were devoid of, or contained little tubulin (Fig. 1A,B; arrows). Distribution of GFP that was not conjugated to tau did not correlate with that of tubulin (Fig. 1A).

**Fig. 2.**
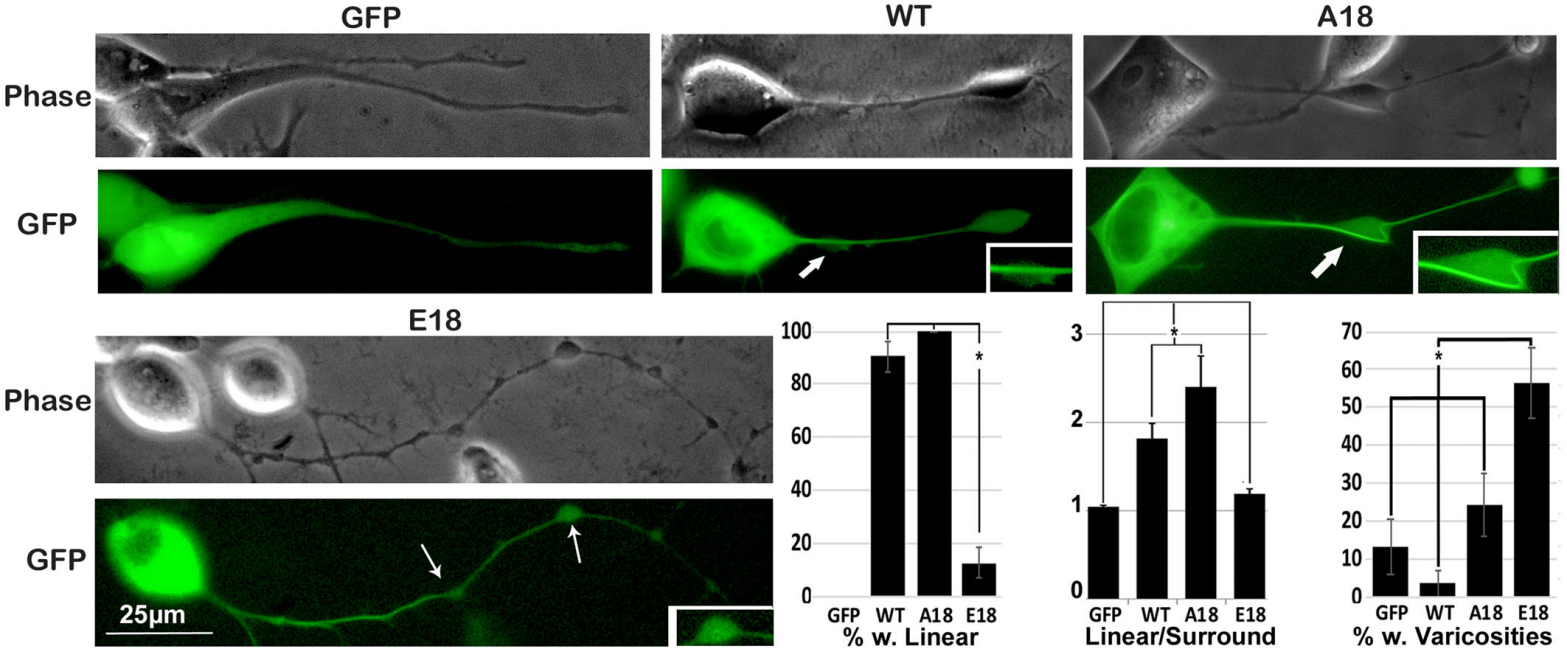
**E18 displays a more diffuse neuritic profile than WT** **or A18.** Panels present representative epifluorescence GFP and corresponding phase-contrast images for cells expressing unconjugated GFP, WT, A18 and E18. As in panel A, visual inspection reveals that WT and A18 displayed a centralized linear profiles within axonal neurites while E18 was more diffuse, extending into the surrounding neuritic cytoplasm (surround) E18 was also dispersed throughout varicosities along axonal neurites (arrows), while both WT and A18 maintained a centralized, linear profile within varicosities. Inserts present one of the indicated varicosities at higher magnification. GFP not conjugated to tau was dispersed throughout neurites with no distinct linear profile. The accompanying graphs, derived from ten cells per each construct derived from three separate experiments (total 30 cells per condition) present the percentage of cells displaying centralized, linear GFP within neurites, the ratio of GFP within linear profiles versus the surrounding neuritic cytoplasm and the percentage of cells with varicosities along neurites. An increased percentage of cells expressing WT and A18 displayed linear GFP within neurites versus those expressing unconjugated GFP or E18 (*P*<0.001 for each). The ratio of linear GFP versus that in the surrounding neuritic cytoplasm was higher in cells expressing WT and A18 versus those expressing unconjugated GFP or E18 (*P*<0.001 for each; one-way ANOVA). An increased percentage of cells expressing E18 displayed varicosities along neurites versus all other conditions. Values represent the mean±standard error. *N*=23 for unconjugated GFP, 32 for WT, 29 for A18 and 20 for E18 for all analyses. All images are at the same magnification.

Differential distribution of tau constructs was further substantiated by comparison of GFP with corresponding phase-contrast images. Both WT and A18 displayed centralized linear profiles within axonal neurites. By contrast, E18 was more diffuse, extending into the surrounding neuritic cytoplasm (‘surround’) within varicosities along axonal neurites, while both WT and A18 maintained a centralized, linear profile within varicosities. GFP not conjugated to tau was dispersed throughout neurites with no distinct linear profile (Fig. 2). A reduced percentage of cells expressing E18 displayed linear profiles, while an increased percentage displayed varicosities (Fig. 2).

These findings are consistent with prior studies demonstrating that E18 was less capable of association with MTs than WT or A18 in cultured embryonic neurons (Rodríguez-Martín et al., 2013), and confirm similar behavior of these tau isoforms in differentiated NB2a/d1 cells.

### Overexpression of tau decreases neurite caliber while only tau pseudophosphorylation increases neurite length

**Fig. 3.**
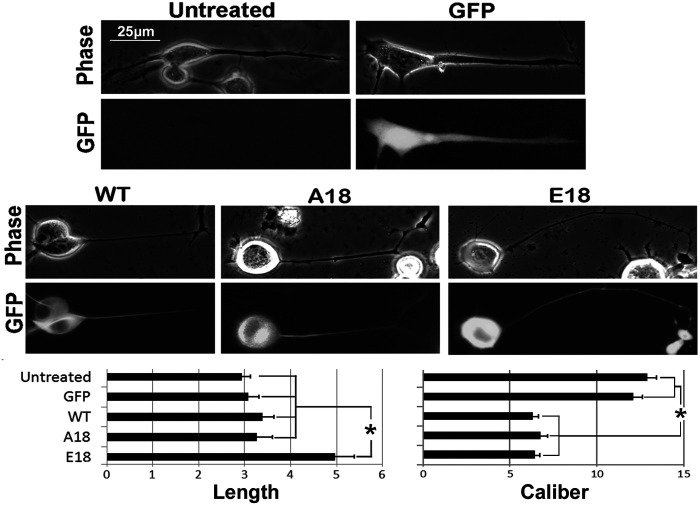
**Pseudophosphorylation of tau increases neurite length while caliber is decreased regardless of phosphorylation state.** Panels present representative images of GFP along with corresponding phase-contrast images of cells transfected with constructs encoding unconjugated GFP, WT, A18 or E18 with neurite outgrowth initiated 48 h later, and observation 24 h after induction of differentiation as described in the Materials and Methods. The accompanying graphs present quantification of length and caliber of neurites from two experiments as described in the Materials and Methods. Untransfected cells (*n*=86) and those expressing unconjugated GFP (*n*=69), WT (*n*=66) or A18 (*n*=56) elaborated neurites of identical length, while cells expressing E18 (*n*=77) elaborated significantly longer neurites (asterisk indicates *P*<0.01). Neurite caliber did not vary between untransfected cells and those expressing unconjugated GFP, but cells expressing WT, A18, or E18 elaborated neurites of reduced caliber (asterisk indicates *P*<0.01; one-way ANOVA). Reduction in caliber was statistically identical for cells expressing each tau construct. All images are at the same magnification. Values represent the mean±standard error.

We previously demonstrated that overexpression of WT tau resulted in elaboration of axonal neurites of increased length and reduced caliber (Dubey et al., 2008). We sought to determine whether pseudophosphorylation of tau or prevention of tau phosphorylation altered the influence of tau on neurite morphology. To accomplish this, cells were transfected with the above constructs and neurite outgrowth was initiated 48 h later, with observation 24 h after induction of differentiation as described in the Materials and Methods. No difference was observed in neurite length among untransfected cells and those expressing with unconjugated GFP, WT or A18. However, neurites elaborated by cells expressing E18 were significantly longer than all other conditions (Fig. 3). Neurite caliber did not vary between untransfected cells and those expressing unconjugated GFP, but cells expressing WT, A18, or E18 elaborated neurites of reduced caliber. Notably, this reduction in caliber was identical for cells expressing each tau construct (Fig. 3).

### Pseudophosphorylation of tau compromises axonal MT and cytoskeletal stabilization

**Fig. 4.**
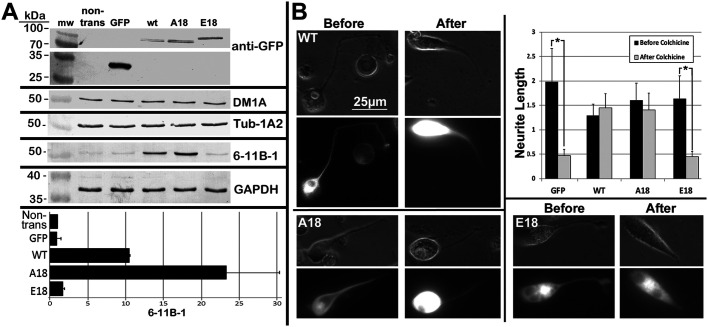
**Pseudophosphorylation of tau compromises axonal MT and cytoskeletal stabilization.** Panel A presents nitrocellulose replicas of supernatants of cells expressing GFP not conjugated to tau, WT, A18 or E18 conjugated to GFP, and from non-transfected cells (non-trans) probed with the indicated antibodies. Migratory positions were ascertained by inclusion of commercial molecular weight markers (mw). Identical levels of GADPH were detected in all lysates, confirming equivalent sample size. Anti-GFP was included to confirm expression of constructs; unconjugated GFP migrated at its anticipated apparent mw of approximately 30 kDa, while GFP-tau constructs migrated at the anticipated mw for tau+GFP; the slightly slower migration of E18 is consistent with extensive pseudophosphorylation of this fusion protein. Total and tyrosinated α-tubulin levels (indicated by DM1A and Tub-142, respectively) were similar among all lysates. By contrast, acetylated α-tubulin levels (indicated by 6-11B-1) was significantly elevated in lysates from cells expressing WT and even more so from those expressing A18. The accompanying graph presents the ratio of 6-11B-1 versus DM1a immunoreactivity for all conditions from duplicate replicas; values represent the average±range. Panel B presents representative cells expressing WT, A18 or E18 before and after treatment with 10^−6^ M colchicine for 2 h. Note that neurites of cells expressing WT or A18 were resistant to retraction while neurites of cells expressing E18 or GFP not conjugated to tau underwent retraction. The accompanying graph presents neurite length in respective somal diameters before and after colchicine treatment from duplicate experiments. Values represent the mean±standard error. Note that neurites of cells expressing WT (*n*=65) or A18 (*n*=35) were resistant to retraction while those of cells expressing E18 (*n*=44) or unconjugated GFP (*n*=9) underwent retraction (asterisks indicate *P*<0.05; one-way ANOVA). All images are at the same magnification.

We examined the impact of expression of these tau constructs on levels of total, non-stabilized and stabilized tubulin. GAPDH immunoreactivity did not vary between samples. Immunoreactivity towards DM1A, which labels total α-tubulin levels, and Tub-1A2, which recognizes tyrosinated tubulin (indicative of MTs that have not undergone stabilization; Kreis, 1987) were identical among lysates from non-transfected cells and cells expressing unconjugated GFP, WT, A18 and E18 (Fig. 4A). By contrast, immunoreactivity towards 6-11B-1, which detects acetylated- α-tubulin, was significantly elevated in lysates from cells expressing WT and even more so from those expressing A18, as compared to untransfected cells and from cells expressing unconjugated GFP or E18 transfected cultures. Moreover, levels of 6-11B-1 in lysates from cells expressing E18 were indistinguishable from nontransfected cells or those expressing unconjugated GFP (Fig. 4A).

Since MTs rich in acetylated tubulin are relatively more stable than total MTs (Black and Keyser, 1987; Cambray-Deakin and Burgoyne, 1987; Gundersen et al., 1987), and overexpression of WT tau reduced axonal neurite stability (Dubey et al., 2008), the selective absence of increased acetylation following expression of E18 observed herein prompted us to examine whether or not expression of E18 or A18 also compromised neurite stabilization. To examine this possibility, we treated transfected cultures with the MT-depolymerizing agent colchicine. Colchicine treatment initially fosters retraction of axonal neurites of NB2a/d1 cells, but neurites develop colchicine-resistance following accumulation of acetylated MTs, MAPs and NFs (Shea and Beermann, 1994; Shea et al., 1990, 1992; Boumil et al., 2018). Axonal neurites elaborated by cells expressing A18 developed colchicine resistance identical to that of cells expressing WT. However, axonal neurites of cells expressing E18 were retracted following colchicine treatment to the same extent as those of cells expressing only unconjugated GFP (Fig. 4B).

**Fig. 5.**
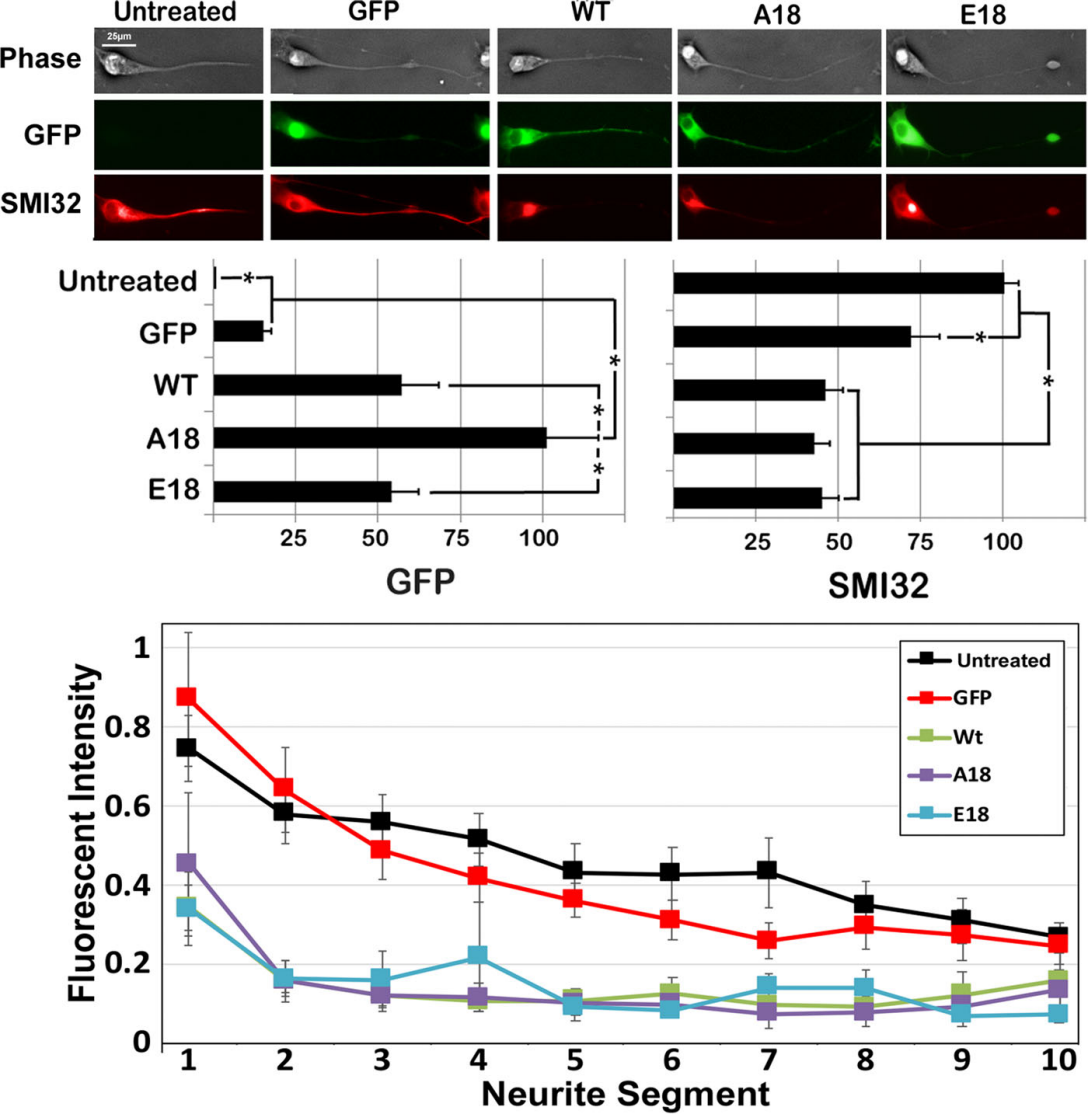
**Overexpression of all tau isoforms reduces neurofilament levels in axonal neurites.** Nontransfected cells and cells expressing unconjugated GFP, WT, A18 or E18 were reacted with monoclonal antibody SMI-32. Representative images are presented for each condition (upper panels); all images are presented at the same magnification. Identical reduction in total levels of SMI32 within axonal neurites was observed following expression of WT, A18 or E18 as compared to levels observed in nontransfected cells or cells expressing unconjugated GFP (middle bar graphs). The number of axonal neurites quantified were a total of 4673 nontransfected cells, 3531 cells expressing unconjugated GFP, 3370 cells expressing WT, 4514 cells expressing A18 and 3834 cells expressing 3834 from triplicate experiments. Asterisks indicate *P*<0.05; one-way ANOVA. The relative distribution of SMI-32 immunoreactivity was also quantified along axonal neurites by dividing neurites into ten equivalent segments and quantifying the percentage of total immunoreactivity within each segment (lower line graph) for a total of 15 cells for each condition from three experiments. Values represent the mean±standard error. For transfected cultures, only cells with a GFP signal were quantified for both GFP and SMI-32.

Prevention of the developmental increase in caliber and colchicine resistance following overexpression of WT tau was correlated with reduction in the developmental accumulation of NFs within axonal neurites in these cells, consistent with the prior demonstration of inhibition of anterograde and increase in retrograde NF transport (Dubey et al., 2008). We therefore monitored steady-state NF content. Expression of all tau constructs reduced overall levels of NFs within axonal neurites to the same extent (Fig. 5).

## DISCUSSION

NB2a/d1 cells are useful to model the early events of axonal cytoskeletal dynamics. Elaboration of axonal neurites is rapid for the first few days following induction of differentiation and is accompanied by accumulation of labile MTs and the intermediate filament vimentin. Axonal neurite outgrowth subsequently slows and caliber expands, commensurate with accumulation of stabilized MTs, MAPs including tau, and NFs (Barry et al., 2012; Cleveland et al., 1991). Neurites then display resistance to retraction following treatment with colchicine, which is derived from one or more of these cytoskeletal elements and potential interactions among them (Shea and Beermann, 1994).

We further examined the role of tau in these dynamics herein by expression of WT tau, tau pseudophosphorylated at multiple sites (E18) and tau unable to be phosphorylated at these sites (A18). Consistent with prior studies, E18 was less capable of association with MTs than WT or A18 and apparently did not induce MT bundling (Rodríguez-Martín et al., 2013). Comparison of E18 with A18 and WT allows further insight into the impact of tau on axonal transport and maturation. All three tau constructs reduced NF levels to an identical extent in axonal neurites. Since E18 does not associate with MTs, this provides further evidence for competition as kinesin cargo with rather than by interference with kinesin processivity as the mechanism by which tau decreases NF content (Dubey et al., 2008). This finding is consistent with inhibition of mitochondrial transport by tau independently of association of tau with MTs (Vossel et al., 2015).

Neurites elaborated by cells expressing WT and A18 developed the anticipated levels of acetylated MTs and resistance to colchicine, despite reduction in NF levels. By contrast, neurites of cells expressing E18 failed to display acetylated MTs or develop colchicine resistance. Failure of E18, which cannot bind to MTs, to confer colchicine resistance to MTs is consistent with the prior demonstration that binding of tau to MTs confers drug resistance (Baas et al., 1994). These data suggest that MT stabilization, rather than NF accumulation, mediates at least the initial development of neurite stabilization. Association of MAPs contributes to MT acetylation (Takemura et al., 1992), which in turn increases axonal transport (Bulinski, 2007). However, recent findings indicate that tau may indirectly contribute to MT stabilization by increasing the length of MTs, followed by MT stabilization by other MAPs (Baas and Qiang, 2019). Since E18 is deficient in MT association, E18 may impair MT stabilization by failure to increase labile MT domains within neurites, which is necessary for ultimate MT stabilization by other MAPs (Baas and Qiang, 2019; Qiang et al., 2018). Observation of slightly higher levels of acetylated MTs in neurites of cells expressing A18 versus those expressing WT is consistent with phosphorylation of some WT tau, and correspondingly less MT association of WT versus A18. The lack of anticipated increase in neurite caliber for cells expressing all tau constructs, but not GFP alone, may derive from reduction in NF content (Shea and Beermann, 1994; Boumil et al., 2015). In this regard, relatively low levels of GFP not conjugated with tau (‘unconjugated GFP’) translocated into neurites, consistent with diffusion of unconjugated GFP versus active transport of GFP-tau conjugates.

Neurites of cells expressing E18 were significantly longer than those of all other cells including non-transfected cultures. Failure of neurites expressing E18 to undergo the anticipated slowing of elongation may derive from lack of stabilization, which may have been augmented by reduction in NF levels within neurites. Notably, while excess tau on MTs inhibits kinesin processivity and can foster detachment of kinesin from MTs, dynein is less sensitive to MT-associated tau and can reverse direction rather than halt or detach upon encountering a MT region saturated with tau (Dixit et al., 2008). It remains possible that, in addition to lack of MT stabilization, continued elongation of neurites by cells expressing E18 is also due in part to less impact on anterograde axonal transport than that induced by WT and A18 (Stamer et al., 2002; Trinczek et al., 1999; Dixit et al., 2008).

While tau is pivotal in neuronal development, it also fosters neurodegeneration. Increased levels of tau are essential for axonal maturation in culture (Dawson et al., 2001; Dubey et al., 2008; Shea et al., 1992; Smith et al., 1995). By contrast, overexpression inhibits anterograde transport, increases oxidative damage, synaptic degeneration and axonal collapse (Stamer et al., 2002; Mandelkow et al., 2003; Thies and Mandelkow, 2007), and disrupts the balance of anterograde and retrograde transport by interfering with both kinesin and dynein (Chaudhary et al., 2018). This interference derives from steric inhibition of motor processivity along MTs (Stamer et al., 2002; Trinczek et al., 1999; Dixit et al., 2008), from tau-induced modification of kinase activity that inhibits kinesin activity (Morfini et al., 2002; Hoeprich et al., 2017), from alteration in kinesin motility by tau phosphorylation (Kanaan et al., 2012) and from tau competing as cargo for kinesin (Dubey et al., 2008).

Imbalance in kinase and/or phosphatase activity that impact tau can underlie neurodegeneration (Johnson and Stoothoff, 2004). Phosphorylated tau is relatively resistant to proteolysis (for review see Johnson, 2006). As such, tau phosphorylation is a pivotal event in formation of paired helical filaments (Hanger et al., 2009). These collective findings underscore the importance of an appropriate balance in tau levels and phosphorylation for normal development and maintenance of the nervous system (Johnson and Stoothoff, 2004).

## MATERIALS AND METHODS

### Cell culture

As in our prior studies, we utilized NB2a/d1 mouse neuroblastoma cells, which elaborate axonal neurites containing most if not all axonal constituents and translocate them into and along neurites via kinesin and dynein. Transfection allowed for expression of multiple forms of tau in the presence of active forms of tau kinases (Dubey et al., 2008). NB2a/d1 cells were maintained in DMEM supplemented with 2 mM glutamine, 10% FBS (Atlanta Biologicals) and antibiotics. Differentiation and elaboration of axonal neurites was induced by treatment with 1 mM dibutyryl-cyclic adenosine monophosphate (dbcAMP).

Cells were cultured on glass-bottom plates (MatTek, Ashland, MA 01721, USA) for live-cell imaging or acid-washed glass coverslips in six-well plates for immunofluorescence analyses. Plates and slides were treated with poly-d-lysine (MW>300,000) for 2 h at room temperature, washed five times with autoclaved double deionized water, and dried overnight under UV exposure. They were then treated with 50 µM laminin for 30 min at 37°C, washed twice with autoclaved double deionized water, and dried for 2 h under UV exposure. For immunoblot analyses, cells were cultured on plastic, tissue culture treated 10 cm plates (CELLTREAT, Pepperell, MA 01463, USA).

### Transfection

Cells were transfected with plasmids which expressed either E18 (Smith et al., 2000), A18 (Cuchillo-Ibanez et al., 2008), or WT full-length human tau (2N4R), all of which were conjugated to EGFP at their C-termini [generous gifts of Diane Hanger (King's College, London, UK) originally obtained from M. Goedert (MRC Laboratory of Molecular Biology, Cambridge, UK)]. E18 features 18 serine/threonine residues mutated to glutamic acid, 16 of which have been identified as GSK3β phosphorylation sites, to mimic constitutive phosphorylation. A18 features mutation of the same 18 serine/threonine residues to alanine to mimic constitutive non-phosphorylation. Additional cultures were transfected with an EGFP-C1 vector with no target gene inserted, which therefore expresses only EGFP (‘unconjugated GFP’).

Cultures were transfected with Polyjet transfection reagent (SignaGen, Rockville, MD 20850, USA) according to the manufacturer's protocol. Levels of GFP were quantified within soma of unfixed cells 24 h after transfection to ascertain relative levels of expression. To monitor the distribution of exogenous tau within established neurites, cultures were differentiated for 48 h prior to transfection, transfected for 4 h and observed 20 h later. For all other experiments, cells were transfected for 4 h before the initiation of differentiation then fixed and imaged or processed for immunoblot analysis after 24 h of differentiation.

### Immunofluorescence

Cultures on coverslips were rinsed twice in phosphate-buffered saline (PBS) and fixed in 4% paraformaldehyde in PBS for 15 min at room temperature. Fixed cultures were rinsed 3× in PBS for 5 min each, blocked with PBS containing 10% goat serum and 0.2% Triton X-100 for 1 h, then incubated with a monoclonal antibody (SMI-32) directed at non-phosphorylated epitopes of the heavy (NF-H) and middle (NF-M) molecular weight neurofilament (NF) subunits (Covance, Princeton, NJ 08540, USA) diluted 1:2000 in PBS containing 2% goat serum and 0.2% Triton X-100 for 2 h at room temperature. Cultures were rinsed three times with PBS and incubated with rhodamine-conjugated goat anti-mouse IgG diluted 1:500 in PBS containing 2% goat serum, 0.2% Triton X-100 for 1 h, and rinsed a final three times with PBS. Coverslips were dehydrated by rinsing with increasing ethanol concentrations (70%, 80%, 95%, 100%, 100%), rinsed twice with xylene, applied to slides with DePeX mounting media (Thermo Fisher Scientific, Waltham, MA 02451, USA) and allowed to dry overnight in the dark.

### Gel electrophoresis and western blot analyses

Transfected cultures on 10 cm^2^ plastic dishes were rinsed in PBS and cells scraped into 200 µl of 50 mM Tris (pH 6.9) containing 20 mM EDTA, 1% Triton X-100, cOmplete™ protease inhibitor cocktail (MilliporeSigma, Burlington, MA 01803, USA), PhosSTOP™ phosphatase inhibitor cocktail (MilliporeSigma) and 1 mM PMSF. Cells were homogenized with a Teflon-glass homogenizer (25 strokes) and centrifuged at 13,100× ***g*** for 15 min at 4°C. The resulting supernatants were heated at 95°C for 5 min in Laemmli buffer at a final protein concentration of 0.75 µg/µl total protein lysate (determined by BCA assay; Thermo Fisher Scientific). A total of 7.5 µg of protein from each was applied 10% polyacrylamide gels in 25 mM Tris, 192 mM glycine, and 0.1% SDS, and electrophoresis carried out at 200 V. Separated proteins were transferred to nitrocellulose in 25 mM Tris containing 192 mM glycine and 10% methanol at 100 V for 1 h at room temperature. Nitrocellulose replicas were blocked in 50 mM Tris buffer (pH 7.6) containing 154 mM NaCl, 0.1% Tween-20, 5% goat serum, and 1% bovine serum albumin for 1 h at room temperature and incubated overnight at 4°C in 50 mM Tris buffer (pH 7.6) containing 154 mM NaCl, 0.1% Tween-20, 2% goat serum plus one of the following antibodies: mouse monoclonal DM1A (directed against alpha-tubulin; 1:2000 dilution; Santa Cruz Biotechnology, Dallas, TX 75220, USA), mouse monoclonal 6-11B-1 (directed against acetylated alpha-tubulin; 1:1000 dilution; Abcam, Cambridge, MA 02139, USA), mouse monoclonal Tub-1A2 [directed against tyrosinated alpha-tubulin (1:1000; MilliporeSigma)], mouse monoclonal anti-GFP (1:500 dilution; Abcam), and anti-rabbit polyclonal anti-GAPDH (1:1000 dilution; Cell Signaling Technology, Danvers, MA 01923, USA). Nitrocellulose replicas were rinsed in 50 mM Tris buffer (pH 7.6) containing 154 mM NaCl and 0.1% Tween-20 (TBST) three times for 5 min and incubated in 50 mM Tris buffer (pH 7.6) containing 154 mM NaCl, 0.1% Tween-20, 2% goat serum plus goat anti-mouse or goat anti-rabbit IgG conjugated to alkaline phosphatase (both diluted to 1:10,000) for 1 h at room temperature. Replicas were then washed twice in TBS containing 0.1% Tween-20, 1× in TBS, and 1× in 100 mM Tris containing 100 mM NaCl, 5 mM MgCl_2_ (pH 9.5). Proteins were visualized by incubation in 100 mM Tris-HCl containing 100 mM NaCl, 5 mM MgCl_2_, 1% 5-bromo-4-chloro-3-indolyl phosphate/ 1.5% nitro blue tetrazolium solution (Abcam).

### Imaging and analysis

Imaging was conducted using a Zeiss Axiovert 200M wide field fluorescence microscope. GFP fluorescence was captured under 50% mercury lamp intensity and 300 ms exposure time. For cultures transfected after 48 h, GFP and SMI-32 fluorescent intensity were independently quantified within soma and the entire axonal neurite (excluding the hillock and growth cone) using ImageJ software (https://imagej.nih.gov/ij/) as described (e.g. Boumil et al., 2018). The relative distribution of SMI-32 immunoreactivity was also quantified along axonal neurites by dividing neurites into ten equivalent segments and quantifying the percentage of total immunoreactivity within each segment (Boumil et al., 2018). GFP was also separately quantified within centralized, linear MT profiles along neurites and within the surrounding neuritic cytoplasm (Boumil et al., 2018. For live-cell images taken after 24 h of differentiation (transfection before differentiation), neurite length and caliber were quantified using the line tool in ImageJ (available at no cost at https://imagej.nih.gov/ij/download.html). Because individual neurites can feature minor variations in caliber along their length, neurite caliber was measured at three distinct, representative portions of the neurite, and averaged to generate a single measurement per neurite. Some cultures were treated with 10^−6^ M colchicine for the final 2 h before imaging (Shea and Beermann, 1994; Dubey et al., 2008); neurite length of these cells were quantified according to their respective somal diameters (Shea and Beermann, 1994; Dubey et al., 2008). Results were analyzed statistically by ANOVA with Fischer's post hoc analyses or Student's two-tailed *t*-test. Since we were utilizing continuous cell lines and multiple cultures, no attempt was made to pre-define a sample size to achieve power.
